# A CRISPR/Cas9-Based System with Controllable Auto-Excision Feature Serving Cisgenic Plant Breeding and Beyond

**DOI:** 10.3390/ijms23105597

**Published:** 2022-05-17

**Authors:** Hao Hu, Fengqun Yu

**Affiliations:** Saskatoon Research and Development Centre, Agriculture and Agri-Food Canada, 107 Science Place, Saskatoon, SK S7N OX2, Canada; hao.hu@agr.gc.ca

**Keywords:** Cas9, cisgenesis, CRISPR, genome editing, plant breeding, vector

## Abstract

Transgenic or genetically modified crops have great potential in modern agriculture but still suffer from heavy regulations worldwide due to biosafety concerns. As a promising alternative route, cisgenic crops have received higher public acceptance and better reviews by governing authorities. To serve the purpose of cisgenic plant breeding, we have developed a CRISPR/Cas9-based vector system, which is capable of delivering target gene-of-interest (GOI) into recipient plants while removing undesired genetic traces in the plants. The new system features a controllable auto-excision feature, which is realized by a core design of embedded multi-clonal sequence and the use of inducible promoters controlling the expression of Cas9 nuclease. In the current proof-of-concept study in *Arabidopsis thaliana* (L.) Heynh., we have successfully incorporated a GOI into the plant and removed the selection marker and CRISPR/Cas9 components from the final product. Following the designed workflow, we have demonstrated that novel cisgenic plant germplasms with desired traits could be developed within one to two generations. Further characterizations of the vector system have shown that heat treatment at 37 °C could significantly improve the editing efficiency (up to 100%), and no off-target mutations were identified in the *Arabidopsis* background. This novel vector system is the first CRISPR/Cas9-based genome editing tool for cisgenic plant breeding and should prove powerful for other similar applications in the bright future of precision molecular breeding.

## 1. Introduction

It is estimated that the global demand for plant crops will increase by 100–110% when compared to the demand in 2005, when the world population reaches 9.6 billion by 2050 [1]. To cope with the great agricultural challenge brought by the rapidly growing population, climate change, and decreased arable land, innovations in plant breeding technology are urgently needed. In modern agriculture, crossbreeding, mutation breeding, and transgenic breeding are the three main strategies for crop improvement. Among them, transgenic breeding generates desired traits by directly introducing beneficial foreign gene(s) into elite background varieties; thus, it can break the barrier of reproductive isolation in crossbreeding and overcome the intrinsic restriction due to the stochastic nature of mutation breeding [2]. Since genetically modified (GM) crops were first introduced in 1994 [3], transgenic breeding has been playing an increasingly pivotal role in modern agriculture. So far, a total of 540 transgenic events in 32 crops have been approved for cultivation worldwide [3]. However, concerns of potential environmental risks, such as chances of gene flow and adverse effects on non-target organisms, and potential toxicity and allergenicity to human beings, have prompted the long and costly regulatory evaluation processes and thus precluded the widespread adoption of GM crops [4]. Alternatively, some new technologies, such as cisgenesis and genome editing, can be utilized to develop improved crops without any exogenous gene, or at least without any disturbance to the sexually compatible gene pool, which is identical to that available for traditional breeding [4,5,6]. By definition, genome editing modifies endogenous gene(s), while cisgenic plants are transformed with gene-of-interest (GOI) derived from the species itself or sexually compatible relative species, and they should be free from other undesired sequences such as selection marker genes (SMGs) [7]. Thus, it is expected that plants generated with these alternative technologies would achieve higher public acceptance as compared to transgenic plants and have faster regulatory approvals [4].

To serve cisgenic plant breeding, scientists have developed some strategies to preclude undesired genetic traces in new crops. First, avoiding using SMGs in transformation is a possible way [8,9,10]. However, the screening for positive transformants is very laborious without the help of SMGs; thus, this method is useful for only a few plant species, i.e., impractical in most species [11]. Second, co-transformation (i.e., transferring GOI and SMG separately on different vectors) is probably the simplest method to obtain SMG-free transgenic crops [12,13,14]. However, this strategy is tedious by design and suffers from major disadvantages rooted in the required segregation process. If the two constructs are integrated into closely linked loci or even the same locus, it could be nearly impossible to segregate them apart from each other. Further, it could not be applied to vegetatively propagated plants, whose genome does not undergo sexual recombination and segregation [11]. Third, the most promising strategy is to create DNA double-strand breaks (DSBs) flanking an SMG using targeted DNA editing tools, especially various site-specific recombination systems, and successful applications have been reported [15,16,17,18,19]. The induction of two DSBs on the same chromosome mainly leads to deletions of the flanked DNA region [20,21]. Still, the current site-specific editing systems all have their shortcomings. Zinc finger nucleases (ZFNs) and transcription activator-like effector nucleases (TALENs) both suffer from construction complexity and lack of robustness in addition to patent restrictions; thus, their use in plants is far from routine [2,22,23]. While for other site-specific recombination systems, such as Cre-*lox* and FLP-FRT, the most prevalent issue is the lack of control in the cleaning process, and some also need major improvements regarding the excessive demand of time and labor to generate clean progeny [11].

As the gold standard in the field of genome editing, the CRISPR/Cas system is a powerful tool that, for the first time in history, has enabled plant breeders to control the specific introduction of targeted sequence variation [2]. This system, consisting of CRISPR (clustered regularly interspaced short palindromic repeat) repeat-spacer arrays and Cas (CRISPR-associated) proteins, is an adaptive immune system in bacteria and archaea, which provides defense against phages and other invasive genetic elements. Based on their signature *Cas* genes and the nature of the interference complex, CRISPR/Cas systems have been divided into six types, and Type II CRISPR/Cas9 from *Streptococcus pyogenes* was the first system shown to accomplish RNA-guided site-specific genome editing in eukaryotic cells [24,25]. In the CRISPR/Cas9 system, the two components, Cas9 nuclease and a single guide RNA (sgRNA), form a Cas9/sgRNA ribonucleoprotein (RNP) complex, and the 20 nucleotides at the 5′ end of the sgRNA direct this complex to create a DSB of DNA at a specific target site following Watson–Crick pairing, which is adjacent to 5′-NGG-3′ protospacer-adjacent motifs (PAMs). The sequence-specific DSBs can be used to introduce a variety of genomic modifications via either of the two DNA repairing pathways in vivo: non-homologous end joining (NHEJ) or homology-directed repair (HDR) [26]. This new system has had numerous successful applications in plants since its debut in 2011 [22,23,27,28,29]. The current advances in CRISPR/Cas9 and its variants and the vast applications in agriculture have been summarized in the review paper by Chen et al. [2], which pointed out a bright future for this system in precision molecular breeding.

In this study, a CRISPR/Cas9-based vector system was devised and tested to serve the need for developing cisgenic plants. With a commonly used procedure of *Agrobacterium*-mediated transformation and selection, novel crop germplasms with desirable traits could be generated within one to two generations, while undesirable genetic traces, including SMGs and/or even the GOI itself, could be cleaned from the final product. More importantly, the cleaning process could be executed in a controlled manner depending on the vector choice.

## 2. Results

### 2.1. Design of the CRISPR/Cas9-Based Vectors with Controllable Auto-Excision Feature

**Figure 1:**
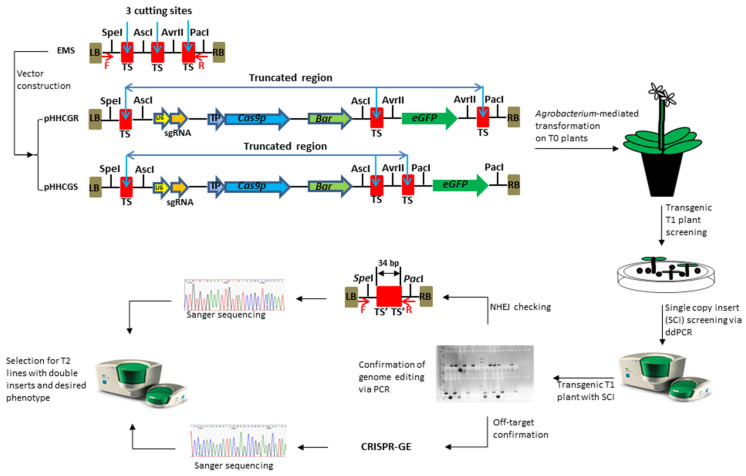
Vector design of a CRISPR/Cas9-based genome editing system with the controllable auto-excision feature and workflow to generate novel cisgenic germplasms. The embedded multi-clonal sequence (EMS) enables the controllable auto-excision. EMS was fully synthesized and cloned into the backbone of the plasmid pYLCRISPR/Cas9Pubi-B [31]. Different components were amplified individually and assembled into different restriction sites depending on their fate in the final product, i.e., removal (sites of AscI and AvrII) or retention (site of SpeI and PacI). LB/RB, T-DNA left/right border; TS, sgRNA targeted sequence (i.e., cutting site by Cas9 protein), originally 23 bp including PAM sequence; TS’, a 17 bp remaining sequence of TS after cutting. The vector pHHCGR and pHHCGS use *eGFP* to demonstrate controllable auto-excision in *Arabidopsis thaliana* (L.) Heynh. where the gene-of-interest is removed (pHHCGR) or retained (pHHCGS) in the final product. *U6*, promoter for sgRNA transcription; sgRNA, DNA template and scaffold sequences for sgRNA transcription; IP, inducible promoter; *Cas9p*, *Cas9* gene modified with plant-optimized codons; *Bar*, phosphinothricin acetyltransferase; *eGFP*, green fluorescent protein gene with an SV40 nuclear location signal (NLS) sequence.

The key feature of this new vector system is the controllable auto-excision of inserted genes. Auto-excision is enabled by the CRISPR/Cas9 components included in each vector, i.e., the sgRNA expression cassette and a plant-codon-optimized *Cas9* gene (*Cas9p*) [30]; while the control over auto-excision activities is realized through two main designs, the embedded multi-clonal sequence (EMS) and the inducible promoters (IPs). The EMS is comprised of three target sites for Cas9/sgRNA cutting and four restriction enzyme (RE) sites for RE cloning (Figure 1). The EMS was synthesized and linked onto the backbone sequence of the plasmid pYLCRISPR/Cas9 Pubi-B [31]. This core sequence, namely pHHCG, was used to construct different series of plasmids via RE cloning (e.g., pHHCGR and pHHCGS series). Depending on the fate of each introduced gene in the end product, each functional component could be inserted into different restriction sites, i.e., the sites of AscI and AvrII for genes to be removed and the sites of SpeI and PacI for genes to be retained. For example, for GOIs intended to stay in the final plant, such as a disease-resistant gene or other beneficial genes functioning throughout plant growth, they could be inserted into site SpeI or PacI; while for genes only supposed to stay and function for a certain period, such as SMGs, site AscI or AvrII is the option of choice.

Using different IPs to drive the expression of *Cas9p* provides more control over the auto-excision process. Initially, four germline promoters, i.e., *CLV3*, *LFY*, *AP1*, and *SDS* [32], and two heat shock protein promoters, *Hsp18.2* and *Hsp81-1* [33] from *A. thaliana* (L.) Heyhn. were cloned and tested in pilot tests. Three of them, *CLV3*, *AP1*, and *Hsp18.2*, showed desirable editing results. The two germline promoters, *CLV3* and *AP1*, are involved in different developmental cues, i.e., early stem cell identity (*CLAVATA3*) and flower meristem identity (*APETALA1*), which could represent an early and a middle/late stage in plant growth, respectively. The promoter *Hsp18.2* could be induced by heat treatment (HT) of 37 °C for 2 h [33]. In this study, the transformed plants showed efficient CRISPR/Cas9-mediated auto-excision when driven by the three selected IPs, and the auto-excision took place under pre-designed conditions (i.e., developmental cues or heat treatment). Therefore, these three IPs could provide adequate control over the editing events for relevant applications.

To design the target site, a 5′-GN(19)NGG format was first determined by the *U6-26* promoter used in the sgRNA expression cassette. A series of 20 bp of random sequences were first BLASTed against the NCBI database to minimize the hit chance with common plant genomes [34], and we have taken extra precautions to avoid hitting with genomes of the *Brassica* family crops since this system was initially designed for canola (*Brassica napus* L.) breeding needs. Those sequences with the least homologous hit from BLAST were tested again with online software CRISPR-GE [35], and the ones with the least off-target predictions against the reference genome of *A. thaliana* (L.) Heynh. (TAIR10) were selected as candidate targets. In pilot tests, three target site sequences with off-target scores of “0” were used to construct three different versions of vectors, and the one with the highest editing efficiency was determined to be the best target site and used in all the vectors of this study.

### 2.2. Undesired Genetic Traces Removed in Cisgenic Arabidopsis

**Figure 2:**
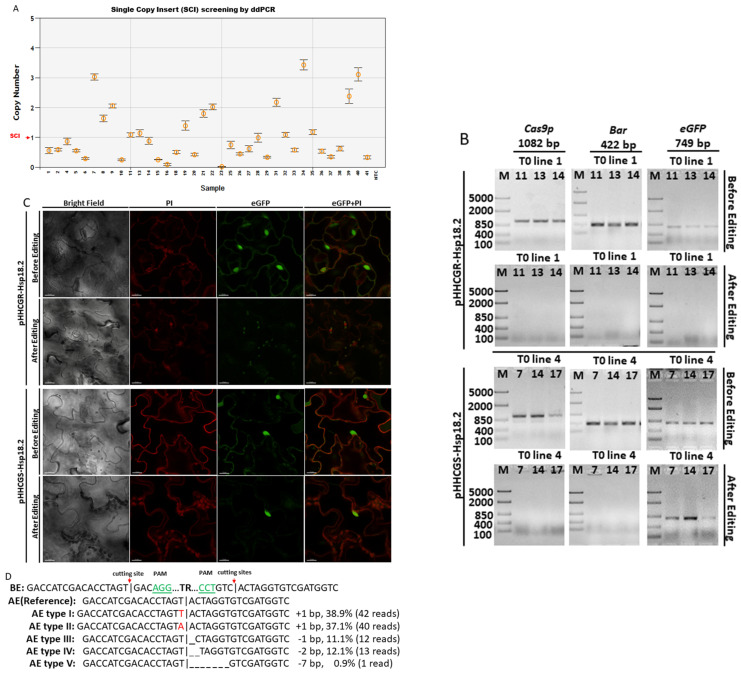

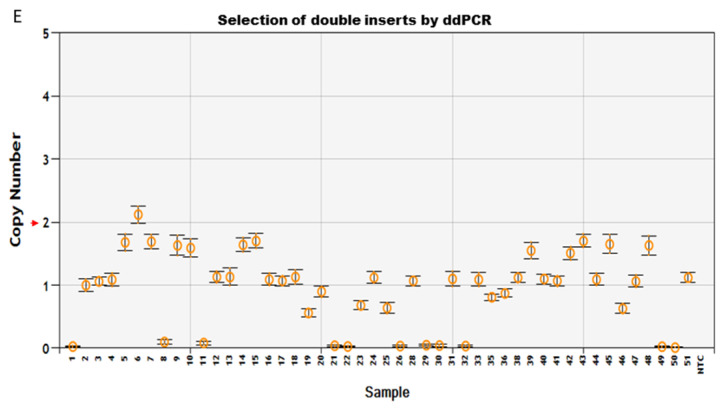
Transgenic components were removed in the final product of *Arabidopsis thaliana* (L.) Heyhn. transformed by pHHCGR−*Hsp18.2*/pHHCGS−*Hsp18.2* vector. (**A**) Screening for T1 lines carrying single copy insert (SCI) of pHHCGR−*Hsp18.2* or pHHCGS−*Hsp18.2* by ddPCR. (**B**) In transgenic T1 lines with SCI, the presence or absence of PCR products of *Cas9p* (1082 bp), *Bar* (422 bp), and *eGFP* (749 bp) showed distinct results in the same plant before and after editing. (**C**) In transgenic T1 lines with SCI, confocal images showed distinct results of eGFP expression in the same plant before and after editing. Bright, bright-field microscopy; PI, propidium iodide, fluorescent dye staining nuclei due to intercalating binding with DNA, excitation wavelength (535 nm) and emission wavelength (617 nm) on confocal microscope; eGFP, eGFP protein mainly accumulated in the nuclear region due to an SV40 NLS added before the *eGFP* coding region, excitation wavelength (488 nm) and emission wavelength (509 nm) on the confocal microscope. Scale bar, 15 µm. (**D**) Sequencing results of PCR products showing the junction sequence after editing and repair via non-homologous end-joining (NHEJ). BE/AE, before/after editing; TR, truncated region; |, cutting site. Three-base PAM sequence highlighted in green, mismatched bases indicated with red font color. (**E**) Selection for T2 lines carrying double inserts of the target gene by ddPCR.

In this study, the green fluorescent protein gene (*eGFP*) was included as a proof-of-concept GOI due to its easy-to-observe phenotype. Following the designed workflow (Figure 1), two series of vectors were first constructed, i.e., pHHCGR-IPs and pHHCGS-IPs. For demonstration purposes, only the results from pHHCGR-*Hsp18.2* and pHHCGS-*Hsp18.2* are presented in Figure 2.

After transformation using the floral dip method, seeds of T0 *Arabidopsis* plants were grown on selection media first. The glufosinate-resistant T1 *Arabidopsis* plants were then transferred from selection media to soil and grown under normal conditions (long-day cycle at 22 °C). At 7 days after transplant, droplet digital PCR (ddPCR) was used to screen for positive transformants with a single copy insert (SCI) of T-DNA (Figure 2A). Only those plants with SCI were used in the following tests to negate the potential effects caused by different copy numbers of inserts. DNA samples from plants with SCI were also checked by PCR to confirm the presence of transgenic components delivered via T-DNA (Figure 2B), while leaf samples were checked by confocal fluorescence microscopy for *eGFP* expression (Figure 2C). The first round of microscopy checking showed that all plants with SCI were positive with *eGFP* expressed at the nucleus location (Figure 2C, before editing). After the first round of sampling, a heat treatment (37 °C for 30 h) was applied to activate promoter *Hsp18.2* and *Cas9p* expression, while the control plants were grown under normal conditions uninterruptedly. One week after the HT, the newly expanded leaf samples were collected and subjected to the second round of PCR and microscopy examination. For the pHHCGR-*Hsp18.2* vector, the PCR results revealed that 15 out of 20 positive transformants with SCI were found to be negative for inserted genes of *Bar*, *Cas9p*, and *eGFP* in the second round of PCR (Figure 2B). The 15 plants also lost eGFP signals in the newly expanded leaves while leaving only red fluorescent signals emitted by propidium iodide (PI) at the nucleus location (Figure 2C). All of the control plants remained positive in the second round of PCR and microscopy checks, indicating no editing/auto-excision happened in the control plants. For the pHHCGS-*Hsp18.2* vector, however, 13 out of 16 positive transformants with SCI turned negative for *Bar* and *Cas9p* in the second round of PCR but not for *eGFP* (Figure 2B). The status of the remaining *eGFP* in those edited plants was also confirmed by confocal microscopy (Figure 2C). The distinct results indicated that the undesired transgenic components such as *Bar* and *Cas9p* could be removed as designed, while the fate of GOI after editing relied on the vector of choice. This experiment also suggested that the editing efficiency of pHHCGR-*Hsp18.2* and pHHCGS-*Hsp18.2* vectors could reach as high as 75% and 81.3% in *Arabidopsis* plants, respectively. 

In order to confirm the editing results, Sanger sequencing was performed on the PCR products of the truncated region using a set of primers from the borders of the T-DNA (primer “F” and “R” in Table S1). As shown in Figure 2D, five types of minor mutations were identified at the joint site compared to the no-error sequence (i.e., reference). This is probably due to the homology of residue sequences at the cutting site on both sides, which could induce errors during NHEJ repair.

Since plants transformed with the pHHCGS-*Hsp18.2* vector showed the best editing results (up to 100% editing efficiency), the seeds of the selected T1 plants with SCI of pHHCGS-*Hsp18.2* and confirmed editing were collected and used for further screening (Figure 2E). The target gene for ddPCR switched from *Bar* to *eGFP* since only GOI remained after editing (see primers in Table S1). The copy number (CN) identified in the T2 population roughly agreed with the theoretical segregation ratio of 1:2:1 for 2 CN:1 CN:0 CN (Figure 2E and Table 1). In addition, unwanted genetic traces (*Bar* and *Cas9p*) were undetected by PCR in T2 plants (up to 100%) (Table 1), which indicated that the auto-excision process was similar in both gametes and somatic cells, hence the heritable cisgenic germplasm [21]. As designed, T2 plants with two copies of GOI and no undesired genetic traces are good candidates as novel cisgenic germplasms.

### 2.3. Characterization of Targeted Editing Controlled by Different IPs and HTs

**Figure 3:**
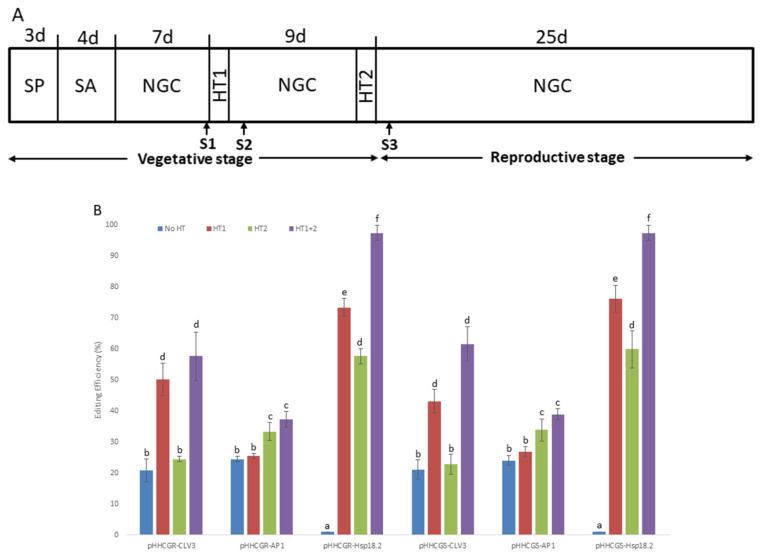

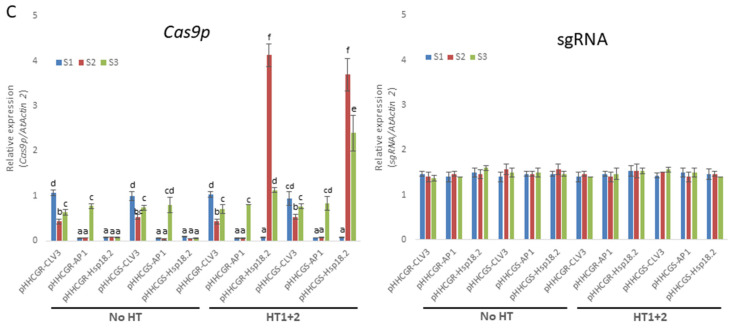
Heat treatments (HT) significantly increase the editing efficiency of all vectors tested in *Arabidopsis thaliana* (L.) Heyhn. (**A**) Schematic representation of the growth conditions of *Arabidopsis* plants exposed to HT. SP, plants selection on plate; SA, soil acclimation; NGC, normal growth conditions (daily 16 h light and 8 h dark, 22 °C during the day and 20 °C at night); HT1 and HT2, first and second HT (37 °C, 30 h); S1/S2/S3, first, second and third sampling of new leaves, S2 and S3 were set at 42 h after HT; d, days. (**B**) Results overview of editing efficiency in different heat treatments and under the control of different inducible promoters. The editing efficiencies of almost all plants tested were significantly affected by different HTs. Error bars represent the range of three replicates. (**C**) RT-qPCR measurement of *Cas9p* and sgRNA expression with different inducible promoters and heat treatment. While sgRNA showed constant and high-level expression in all plants tested throughout the monitored period, the expression levels of *Cas9p* showed quite distinct variations among vectors with different IPs and HTs. Error bars represent the range of three biological replicates. Different letters above bars denote statistical differences.

As depicted in the schematic diagram (Figure 3A), different HTs were applied on T1 *Arabidopsis* plants transformed by six vectors with different IPs. The results showed that the control plants (i.e., treatment “No HT”) transformed by vectors with promoters *CLV3* and *AP1* showed a moderate editing efficiency (about 18.9–24.5%), while all of the control plants transformed by vectors with *Hsp18.2* were not edited at all (i.e., 0%). This may represent the natural performance of the vectors without other stimulations (such as HTs). After HTs were applied, the plants transformed by vectors with promoter *CLV3* showed comparable editing efficiency (ranging from 50.2% to 57.6%) in “HT1” and “HT1+2”, which were significantly higher than that of “HT2”. On the other hand, plants transformed by vectors with promoter *AP1* showed significantly higher editing efficiency (ranging from 33.3% to 37.3%) in “HT2” and “HT1+2”, while “HT1” was not associated with a difference in editing efficiency compared with the control plants (treatment “No HT”). Regarding the plants transformed by vectors with promoter *Hsp18.2* (i.e., pHHCGR-*Hsp18.2* and pHHCGS-*Hsp18.2*), the editing efficiency of both groups followed the same pattern of a stepwise significant increase in the order of “HT2”, “HT1”, and “HT1+2”, with almost complete editing in treatment “HT1+2” (95% to 100% in different replicates). A significant impact of HT on editing efficiency was observed in almost all plants tested (Figure 3B).

The difference in editing efficiency among different HTs may well likely be attributed to the consorted effect between each HT and the expression of *Cas9p* and sgRNA, which warrants the monitoring of their expression levels by RT-qPCR. In all the cases studied, the expression level of sgRNA driven by the *AtU6-26* promoter was about 1.5- to 1.6-fold that of the high-level expression gene *AtActin2* (Figure 3C). The sgRNA levels were roughly constant across vectors with different IPs and remained unaffected by either one or two rounds of HT (Figure 3C). The constant and high-level expression of sgRNA throughout the monitored period suggested that the abundance of the sgRNA was not a limiting factor for gene editing in all the cases analyzed. On the contrary, the expression levels of *Cas9p* showed quite distinct variations among vectors with different IPs and HTs. The expression levels of *Cas9p* driven by promoter *CLV3* (pHHCGR-*CLV3* and pHHCGS-*CLV3*) started at about 1.1-fold that of *AtActin2* and dropped to 0.5- to 0.7-fold at later stages (S2 and S3). For promoter *AP1* (pHHCGR-*AP1* and pHHCGS-*AP1*), the expression of *Cas9p* started at basal levels (about 0.06- to 0.08-fold that of *AtActin2* at S1 and S2) and jumped to 0.7- to 0.8-fold that of *AtActin2* at a later stage (S3). In plants transformed with pHHCGR-*CLV3* (or other vectors with promoter *CLV3*/*AP1*), similar expression levels of *Cas9p* were detected between the two treatments of “No HT” and “HT1+2” (Figure 3C), which indicated that the amount of Cas9p nuclease in these plants was not affected by two rounds of HT. Therefore, the significantly increased editing efficiency in “HT1+2” compared with “No HT” may likely be attributed to the increased activity of Cas9p nuclease at 37 °C [36] (Figure 3B). The promoter *Hsp18.2* could be activated by HT; thus, after the first HT, a peak level (about 4-fold that of *AtActin2*) of *Cas9p* was detected at S2. While the expression level dropped to about 1.1-fold that of *AtActin2* at S3, it was still much higher compared to the constant basal levels (about 0.08-fold that of *AtActin2*) detected in plants of “No HT” (Figure 3C).

### 2.4. No Off-Target Mutations Detected

For CRISPR/Cas9 applications in plants, a low frequency of off-target mutations has been reported [2,37]. When selecting the sgRNA target sequence with CRISPR-GE, extra precaution was taken to find the best possible one. However, in off-target prediction by CRISPR-GE, potential off-target loci with low scores still existed. For recognition specificity and cleavage efficiency of Cas9 nuclease, PAM and the “seed sequence” (12 nucleotides adjoining the PAM) of the target site play critical roles. Therefore, based on these critical sequences and off-target scores [38], nine putative sites were selected and examined by PCR and Sanger sequencing using specific primers designed with PrimerDesign-A [39]. In all of the plants tested, no mutations were found at those putative off-target sites (Table 2), which indicated that the targeted editing induced by these vectors is highly specific, at least in the *A. thaliana* (L.) Heyhn. background.

## 3. Discussion

For agricultural scientists, it is quite understandable that an accurate operation on a limited number of genes using DNA recombination techniques would achieve more specific results than those created by random mutation or genome-wide exchange/recombination. Moreover, there are intractable barriers (such as sexual incompatibility) or inaccessible species (such as asexually reproduced crops) in crossbreeding that do not allow for traditional breeders. Thus, GM crops are the most promising future for humanity in terms of food security. However, transgenic breeding is not a perfect solution. The exogenous gene from other organisms such as bacteria or unrelated plants could disturb the gene pool of the recipient plant, which has been inherited as such for a history maybe longer than humans. Additionally, the insertion sites of the exogenous genes into the recipient genome are random. Both factors may have unpredictable side effects. Hence, GM crops have stirred up considerable concerns over the safety issue in the general public, although no definite incidents have been confirmed by the scientific society. As a result, governments worldwide have implemented strict regulations over the deliberate release of GM crops into the environment.

To address the concerns of the public and governing authorities, scientists have developed a new route of cisgenesis, which emphasizes the use of GOI derived from the same species or sexually compatible relatives of the recipient plant and the cleaning of insertion traces in the end product [7,40]. This new route addresses the major issues of GM crops like gene pool disturbance and undesired residue in new crops, thus has received support from peer scientists [4,5,6,19,41,42,43]; however, the debate over loosening regulations on cisgenic plants never ceases since the debut date [44,45,46]. From the scientific point of view, the European Food Safety Authority (EFSA) Panel has concluded that similar hazards can be associated with cisgenic and conventionally bred plants [47]. Nevertheless, based on the facts that the introduced GOI is naturally present in the same gene pool and that the new cisgenic plants are free of any undesired sequences, the new concept of cisgenic plants is likely to be more acceptable to the general public than transgenic plants. This prediction has been confirmed by numerous surveys conducted in the USA and the EU. For example, a nationwide survey showed that 52.7–77.3% of the US customers would eat a cisgenic vegetable (depending on the number of genes), which is significantly higher than the 17.3–25.7% of participants who would eat a transgenic vegetable containing genes from non-plant source [48]. The 2010 Eurobarometer 73.1 Survey conducted in the EU used the cisgenic apple as an example, which received higher support (55%) than transgenic apples (33%) in all EU countries, with majority support in 24 countries [49]. The organizers also concluded that the public of the EU might perceive the ‘morally more acceptable’ cisgenic plants as an example of the so-called ‘second generation’ of GM crops, which have better ratings in customer benefits, environment safety, and ‘naturalness’ [50]. To serve the bright future of cisgenic plants, we have devised this new system to facilitate the cleaning of undesired genetic traces. Further, the inserted GOI itself could also be eliminated from the end product should it becomes a concern in regulators’ eyes. In a word, this new system provides an effective solution to manage the fate of each inserted component, which could serve the need for breeding cisgenic plants and other similar applications.

The key feature of controlling the fate of inserted sequences is realized by the EMS, which comprises three pre-designed CRISPR/Cas9 sites for targeted editing and two types of restriction enzyme sites for each functional component (Figure 1). This mosaic design enables end-users more control over the fate (i.e., retention or removal) of each functional component in the end product, and breeders could choose which restriction site for their GOI(s) or even potentially make rearrangement of other components using common cloning techniques. In this study, the pHHCGR and pHHCGS vector series were designed to integrate cisgenic GOI into recipient plants via *Agrobacterium*-mediated transformation while under the control of different inducible promoters to perform multiple CRISPR/Cas9-based targeted genomic cuttings to remove undesired genetic traces from the final product. By design, the only difference between pHHCGR and pHHCGS series of vectors is that, if using pHHCGR, GOI is also considered an undesired sequence and removed, and no other significant differences (e.g., editing efficiency or *Cas9* expression pattern, etc.) between them were observed in this study. In a broader field, this system provides a vector platform for gene integration and auto-cleaning of genetic traces in a controlled manner afterward; thus, it could be used beyond the scope of cisgenic crop development.

As shown in Figure 3B, the editing efficiencies of different vectors are generally adequate (around 20% or above), and it could achieve almost complete editing (i.e., editing efficiency as high as 100%) when using the right combination of inducible promoter and heat treatment. Therefore, the fate of each inserted component is fully controllable using this vector system. Additionally, the cleaning process of each undesired genetic component could be accomplished automatically along with the plant growth or with simple heat treatment. To gain a better understanding of the editing activity, different inducible promoters controlling the expression of *Cas9p* were characterized in this study, and the results showed that the pattern of *Cas9p* induction and the followed editing events were generally as designed. For example, the promoter *Hsp18.2* was induced after HT, while promoters *CLV3* and *AP1* were induced at the relevant developmental stages, respectively (see *Cas9p* expression profile in Figure 3C). In practical applications, the *Arabidopsis*-originated inducible promoters tested in this study may not be ideal for certain host plants or specific needs; hence, new promoters could be tested for the replacement of current promoters by users. Heat treatment at 37 °C has been reported to be able to increase the gene-editing activity of the CRISPR/Cas9 system [36], and it was proven effective in boosting the editing efficiency of all the vectors tested in this study (see the significant differences between “No HT” treatment and treatments with HT in Figure 3B). Therefore, HT is a valuable tool for achieving better editing results in practical use. The mechanism behind this boosting effect by HT is likely due to the increased activity of Cas9 nuclease because the *Cas9* expression level is not significantly increased by HT for the four vectors with promoter *CLV3* or *AP1* (Figure 3C). Off-target rate is a critical aspect of evaluating CRISPR/Cas9-based applications [2], and we have demonstrated that the current vector system has such a low off-target rate that no mutations were detected against an extended list of potential off-target sites. This advantage could largely be attributed to the carefully designed target sequence of sgRNA in this study, which has yielded the highest specificity and lowest off-target score in the *Arabidopsis* background [2,39]. The same target site also yields high specificity and undetectable off-target mutations in the canola background (unpublished data). Still, when applied in plants other than *Arabidopsis*, a quick check on the specificity and off-target score of the sgRNA sequence against the relevant genomic background is highly recommended.

With the high performance of this system, a streamlined workflow was devised to develop cisgenic germplasms with homozygous GOI for sexually propagated plants, such as *Arabidopsis*, and a successful application of this workflow could generate novel cisgenic plants within two generations (Figure 1).

Step 1, Vector construction. To choose the appropriate vector, users with specific breeding needs first need to pick between pHHCGS and pHHCGR series of vectors depending on the fate of the GOI in the final product, and then pick one of the three IPs depending on how you want the auto-excision to take place.

Step 2, Transformation and initial screening. After *Agrobacterium*-mediated transformation, positive T1 transformants with SCI of T-DNA should be selected using ddPCR.

Step 3, Editing confirmation. With controlled induction and timing, T1 plants that undergo successful editing/cleaning could be selected with PCR and confirmed with sequencing.

Step 4, Selection for cisgenic germplasm. Further screening and selection will be conducted in the T2 progenies, which will identify progenies with homozygous GOI, no undesired genetic traces, and exclude those with abnormal phenotypes.

For asexually propagated plants, which can produce identical clones of the parent plant, the workflow could be modified at “Step 4”, where the screening and selection process should be conducted on the vegetatively propagated material (leaf-cutting, stem-cutting, etc.) to track the desired trait. In this case, cisgenic germplasm could be developed in T1 generation for vegetatively propagated plants. However, to eliminate any unpredicted position effect caused by random insertion during transformation, this screening and selection process could continue for several generations while GOI and desired phenotypes become stabilized. The whole workflow is straightforward, fast, and not labor-intensive, and it should be easily applied to common crops where *Agrobacterium*-mediated transformation is available.

## 4. Materials and Methods

### 4.1. Plant Materials and Growth Conditions

The *A. thaliana* (L.) Heyhn. ecotype Col-0 was used in this study. Seedlings were germinated on Murashige–Skoog (MS) nutrient agar medium. After being transferred into the soil, the seedlings were grown under long-day conditions (16 h light and 8 h dark at 22 °C). In experiments involving HT, each HT session was 37 °C for 30 h, after which regular long-day conditions were applied (Figure 3A).

### 4.2. Construction of Vectors

The target sequence containing a 20 bp of sgRNA (5′-GACCATCGACACCTAGTGAC-3′) and a 3 bp of PAM (5′-AGG-3′), which could be recognized and cut by the sgRNA/Cas9 ribonucleoprotein complex, was selected with a web-based software CRISPR-GE [35,39]. In off-target predictions, the selected target sequence obtained the lowest score when searched against reference genomes of *A. thaliana* (L.) Heyhn. (TAIR 10). A 105 bp of EMS containing 3 repeats of the target sequence and 4 unique restriction sites (Figure 1) was designed as a core sequence. The EMS and all primers/probes used in this study were synthesized by Integrated DNA Technologies (Coralville, USA). The EMS was first stitched onto the backbone of plasmid pYLCRISPR/Cas9Pubi-B [30] by replacing the original sequence between the left border (LB) and the right border (RB) using an In-Fusion PCR Cloning Kit (Clontech, San Jose, CA, USA). This initial step formed the core structure of vector series, namely pHHC(is)G(enesis). Each functional component, i.e., *Cas9p* gene, sgRNA cassette, SMG, and GOI, was constructed individually using the In-Fusion PCR Cloning Kit (Clontech). For example, the *AtU6-26* promoter was amplified from Construct #1 [51] and used to drive the expression of selected sgRNA. Three promoters, *CLV3* of *CLAVATA3* (*AT2G27250*), *AP1* of *APETALA1* (*AT1G69120*), and *Hsp18.2* of *Heat Shock Protein 18.2* (*AT5G59720*), were amplified from *A. thaliana* (L.) Heyhn. Col-0 and used to drive the expression of the *Cas9p* gene in different constructs, respectively [32,33]. The selection marker gene, *Bar*, driven by an enhanced CaMV 35S promoter, was amplified from plasmid pYLCRISPR/Cas9Pubi-B [30] and placed after the *Cas9p* gene. As a proof-of-concept, the *eGFP* gene was amplified from Construct #2 [51] and used as the GOI in the vector series of pHHCGR or pHHCGS. All vector design and construction work were performed with SnapGene v5.0 (Insightful Science, San Diego, CA, USA, available at snapgene.com accessed on 20 April 2022).

### 4.3. Transformation and Identification of Transformants

The constructed plasmids were electroporated into *Agrobacterium tumefaciens* GV3101 individually. *Agrobacterium*-mediated transformation was performed using the floral dip method [52]. After transformation, the seeds of T0 *Arabidopsis* plants were collected and used to screen for positive transformants on MS agar plates containing 40 μM of glufosinate ammonium. The glufosinate-resistant *Arabidopsis* plants were then transferred to soil and used for subsequent experiments.

### 4.4. Post-Transformation Tests with PCR, ddPCR, and Confocal Fluorescence Microscopy

Genomic DNA was extracted from the newly expanded leaves using the DNeasy Plant Mini Kit (Qiagen; Toronto, ON, Canada). Genomic DNA samples were used as templates in conventional PCR and ddPCR reactions. Conventional PCR was used to confirm the presence or absence of inserted genes, i.e., selection marker gene (*Bar*), CRISPR/Cas9 components (*Cas9p*), and gene-of-interest (*eGFP*). The PCR reaction mix and PCR protocol followed instructions of Taq DNA Polymerase purchased from New England Biolabs (Ipswich, MA, USA), and the amplification was performed on the Veriti 96-Well Fast Thermal Cycler (Applied Biosystems, Mississauga, ON, Canada). A ddPCR system was used to assess the copy number of inserted T-DNA in the transformed plants. The *Actin2* gene of *A. thaliana* (L.) Heyhn. (*AtActin2*) was used as a reference, and *Bar* or *eGFP* gene was used to evaluate the copy number of insertions. The ddPCR reactions and data analysis were performed with the QX200 Droplet Digital PCR System (Bio-Rad, Hercules, CA, USA; QX Manager Software Standard Edition v1.2) following the manufacturer’s instructions.

The leaves from the positive *Arabidopsis* plants (confirmed by PCR) were stained with PI and imaged on a Zeiss LSM710 (Carl Zeiss Ltd., Jena, Germany) with 10 × 40 magnification. PI-stained nuclei and eGFP were imaged using a 535-nm and 488-nm excitation wavelength, respectively.

### 4.5. Characterization of Editing Activity Controlled by IPs and HTs

T1 *Arabidopsis* plants carrying SCI were used in a series of experiments to characterize the editing activity controlled by different IPs and HTs. For each vector tested, three T0 lines served as three biological replicates, and about twenty T1 plants of each T0 line received 4 different treatments as follows. After transplanting and soil acclimation, seedlings were either continuously grown under normal conditions (long-day cycle at 22 °C) or exposed to HTs (long-day cycle at 37 °C for 30 h). The first HT (HT1) was applied 7 days after soil acclimation, and the second HT (HT2) was applied right before entering the reproductive phase, corresponding to the developmental cues of *CLV3* and *AP1*, respectively (Figure 3A). Thus, except for the control plants growing without HT (“No HT”), other plants received either one (“HT1”, “HT2”) or two HTs (“HT1+2”).

After each HT, the plants were allowed to recover for 42 h at 22 °C before sampling for new leaves, and the 3 sampling time points were selected to represent the early stage before HT (S1), 42 h after HT1 (S2), and 42 h after HT2 (S3). Genomic DNA was isolated from each plant and used in PCR and ddPCR tests. The total RNA was isolated from the pooled new leaves of all positive plants of the same transgenic line (about 20 plants) and used in RT-qPCR to monitor the expression patterns of *Cas9p* and sgRNA. Reverse transcriptions for *Cas9p*, *AtActin2*, and sgRNA were carried out with 1 μg of total RNA using a mixture of specific primers, and then the second step of qPCR was performed with each specific primer set (Table S1) on the StepOne Plus Real-Time PCR System (Applied Biosystems; StepOnePlus^TM^ Software v2.3). *AtActin2* was used as the internal control to calculate the relative expression levels of *Cas9p* and sgRNA at different time points (Figure 3C).

To simplify the analysis, only T1 plants with SCI were included in the calculations, which should negate the possibility that different copy numbers of T-DNA account for the results obtained among different treatments. To calculate editing efficiencies, each plant was tested twice, one at an early stage before HT (S1 in Figure 3A) and the other one at a late stage after vegetative growth and HT (S3 in Figure 3A). When a plant was confirmed to be positive for SCI in the test of S1 and was tested negative for removable genes in the test of S3, then the plant was considered to be an “edited plant”. The editing efficiency was the result of all the edited plants (from S3) divided by the total positive transformants confirmed (from S1) (Figure 3B).

### 4.6. Sanger Sequencing

Targeted gene editing and potential off-target mutations were confirmed by PCR and Sanger sequencing. Six edited plants of each vector were examined. After PCR with specific primers (Table S1), amplicons of expected sizes were cloned into the PCR 2.1 Vector using the TA Cloning Kit (Invitrogen, Waltham, MA, USA). For each targeted editing site, at least nine individual clones were sequenced. The sequence analysis was performed with SnapGene v5.0 (Insightful Science).

### 4.7. Statistical Analysis

One-way ANOVA with post-hoc Tukey HSD tests was performed using the R package 3.6.1 (http://www.R-project.org/) (accessed on 30 May 2021) for variance and significance analysis with a significant level of *p* = 0.05.

## 5. Conclusions

A novel CRISPR/Cas9-based vector system with a controllable auto-excision feature was developed for cisgenic plant breeding for the first time. It also provides a powerful tool for other similar applications in the bright future of precision molecular plant breeding.

## Figures and Tables

**Table 1:** Confirmation of target genes in T2 progenies of selected *Arabidopsis thaliana* (L.) Heyhn. T1 lines carrying single copy insert of pHHCGS-*Hsp18.2*.

T1 Lines	T2 Plant Counts				
	Copy Number of *eGFP* (by ddPCR)			Gene Presence (by PCR)	
	2 CN	1 CN	0 CN	*Bar*	*Cas9p*
4–7	12	25	11	0	0
4–14	10	26	12	1	1
4–17	12	23	13	0	0

**Table 2:** Confirmation results in potential off-target mutations.

Chromosome	Position	Sequence ^1^	Off-Target Score ^2^	Gene	Number of Mutations
1	22260438	CACCATCGACACCAAGTAAA **ATG**	0.006	*AT1G60410*	0
1	3107483	GACCATCAGCACCAAGAGAC **AGC**	0.005	*AT1G09590*	0
1	3136508	GACCATCAGCACCAAGAGAC **AGC**	0.005	*AT1G09690*	0
3	1155003	CAACAGCGACACCTTGTGAC **AGC**	0.002	*AT3G04350*	0
2	12697016	GACCAACCACACCTATTGCC **AGC**	0	*AT2G29690*	0
5	24455623	GAGCATCGACACCGCTTGAC **AAG**	0	*AT5G60790*	0
3	22672488	GA**A**CAT**T**GACA**G**CTA**C**T**C**AC **AGG**	0	*AT3G61250*	0
2	13196454	GACC**T**TC**T**ACA**G**CTA**T**TGA**A AGG**	0	*AT2G31010*	0
5	22363439	GACCATC**A**A**G**A**G**CT**TT**TGAC **AGG**	0	*AT5G55100*	0

^1^ Three-base PAM sequence highlighted in green, mismatched bases indicated with red font color. ^2^ Off-target scores are cited from CRISPR-GE [35].

## Data Availability

All of the data generated or analyzed during this study are included in this published article and its Supplementary Information Files. The vectors are available from the corresponding author on reasonable request.

## Supplementary Materials

The following supporting information can be downloaded at: https://www.mdpi.com/article/10.3390/ijms23105597/s1.

## Abbreviations

ANOVA	analysis of variance;
*AP1*	Promoter of *Arabidopsis* gene *APETALA1* (flower meristem identity);
BLAST	Basic local alignment search tool;
*CLV3*	Promoter of *Arabidopsis* gene *CLAVATA3* (early stem cell identity);
CN	Copy number;
CRISPR	Clustered regularly interspaced short palindromic repeats;
Cas	CRISPR-associated protein;
*Cas9p*	*Cas9* gene modified with plant-optimized codons;
Cre-*lox*	site-directed recombination system including Cre recombinase and *Lox* sequence;
ddPCR	Digital droplet polymerase chain reaction;
DSB	Double-strand break;
*eGFP*	Enhanced green fluorescent protein;
EMS	embedded multi-clonal sequence;
GM	Genetically modified;
FLP-FRT	site-directed recombination system including recombinase flippase (FLP) and flippase recognition target (FRT);
GOI	gene-of-interest;
HDR	Homology-directed repair;
Hsp	Heat shock protein;
HT	Heat treatment;
IP	inducible promoter;
NHEJ	Non-homologous end joining;
PAM	protospacer-adjacent motif;
PCR	Polymerase chain reaction;
PI	propidium iodine;
RE	Restriction enzyme;
RT-qPCR	Quantitative reverse transcription polymerase chain reaction;
SCI	Single copy insert;
sgRNA	single guide RNA;
SMG	Selection marker gene;
TALENs	Transcription activator-like effector nuclease;
T-DNA	transferred DNA;
ZFNs	Zinc finger nucleases
