# Supplementary material for: A CRISPR/Cas9-Based System with Controllable Auto-Excision Feature Serving Cisgenic Plant Breeding and Beyond
Source: Int J Mol Sci. 2022 May 17;23(10):5597. doi: 10.3390/ijms23105597 (PMC9143149; doi:10.3390/ijms23105597)
Supplement: Supplementary file 1 [file ijms-23-05597-s001.zip › ijms-1715800-supplementary.pdf]

Table S1. Primers and probes used in this study.

Primer name	Sequence (5′-3′)	Note
**F**	GCAAGTTAAAATAAGGCTAGTCCG	PCR confirmation and sequencing after editing; 189 bp of amplicon
**R**	GGATGTGCTGCAAGGCGATTAAG	
**AtActin2-P**	HEX- CCCGATGGGCAAGTCATCACGATT -BHQ1	Insert copy number screening by ddPCR; *AtActin 2* as reference gene
**AtActin2-F**	GCAGGAGATGGAAACCTCAA	
**AtActin2-R**	CTTCTGGGCATCTGAATCTCTC	
**Bar-P**	6-FAM-ACGGTCAACTTCCGTACCGAGC-BHQ1	Single copy insert (SCI) screening by ddPCR; *Bar* as target gene
**Bar-F**	GTCAACCACTACATCGAGACAAG	
**Bar-R**	GCGACGAGCCAGGGATA	
**eGFP-P**	6-FAM- AGCCACAACGTCTATATCATGGCCG - BHQ1	Insert copy number screening by ddPCR; *eGFP* as target gene
**eGFP-F**	GCTGAAGGGCATCGACTT	
**eGFP-R**	TGCCGTTCTTCTGCTTGT	
**BarPTC-F**	CACCATCGTCAACCACTACATCG	Post-transformation confirmation for *Bar* gene by PCR; 422 bp of amplicon
**BarPTC-R**	AACCCACGTCATGCCAGTTC	
**Cas9pPTC-F**	GGTGCCTAATGAGTGAGCTAACTCAC	Post-transformation confirmation for *Cas9p* gene by PCR; 1082 bp of amplicon
**Cas9pPTC-R**	CGTCCACTCCTGCGGTTC	
**eGFPPTC-F**	GGAGAGAACACGGGGGAC	Post-transformation confirmation for *eGFP* gene by PCR; 749 bp of amplicon
**eGFPPTC-R**	GTACAGCTCGTCCATGCCG	
**Cas9RT-F**	CGCTCAGATTGGAGATCAGT	Quantification of *Cas9p* gene expression by RT-qPCR; from Ma et al., 2015
**Cas9RT-R**	CCTGGTGGTGCTCGTCGTAG	
**sgRNART-F**	GACCATCGACACCTAGTGACG	Quantification of sgRNA expression by RT-qPCR; from Ma et al., 2015
**sgRNART-R**	CGACTCGGTGCCACTTTTTCAAGTTG	

Sequences

1. **Embedded multi-clonal sequence (EMS) 105 bp**

agtcACTAGTGACCATCGACACCTAGTGACAGGGGCGCGCCGACCATCGACACCTAGTGACAGGCCTAGGCCTGTCACTAGGTGTC
GATGGTCTTAATTAAgtac

2. **Inducible promoter sequences:**

Hsp18.2 720bp

ATGGTCATTTCTTCTGGTTCAAGCATGACATGAACAGGCAATAAATAAGTTGAGATTTTGATCACAGTAACTGATACTTGAATCG
AATCATTTAGATTTTTTTTTTTTTTAGTTTACTTGTTTAGTAAATATGTTGTCTATGTTTGTCACAAAAACGTGGCTCAGTTCTTGTAT
ATATGGAGACAAAAAAATCCATTAAAAGATTGTTGACATTCTCGGAAATTTAGTGCCAACTGTTATTGCGAGAACTTACTATAGT
TTTCCTTTGGCGAAAAGCTAATAATCTTAAATCTTGATTTTGTCCTCTTTTCTCTGAGTTAGATTTTCTTAAATTCCACTTCCGACCT
ATTAAGAAATGGGCTTTTGCAAAGAAGATCCGCTTCACTGAGCCCGTATCTCGAAGAGGATAATACAACAACAAAGCAAAACG
GCACGTAGTTTTAATTGTAACCAAGGATTGCATTTCGGTCTTGTTTCAACAAACGAAACTTCCTGAAATGCCAAGAAAAATCTGG
TCATTTCACCACAGTGATCATTGTGTATGTGTTCTAAAGACTCCAAGCGAAGGTTTTAGAAAAAGGAGCATTTTCTATTCTATTCA
AGAAACTCGAAGAACATTCTCTCTTCATCCTCTAACTTCCCTATAAATATGTCCTTTGCTAATCAGATCAAATCAGCAGGAAAATC
AAGAACCAAAAGTCTCCCGAAAAGCAACGAACA

CLV3 1444 bp

CGGATTATCCATAATAAAAACAAAACTAGATACATTTTCTAAGTACATTAACACATAAGAATATCATTTTTGTACTACCAAAAAA
AAAGTAAAAACTTATGTCCATGGTATTATTATAATTGGTGGTAAATCAGTAAATTAGTTGTTAGCAAAAAAATAAAATTTAGTTA
AAAAGTAGTGGCACCTTATTGGCCCAAAAGAGTAACGAAGCAAAACGGAATCTGAAAACTGTTAAAACTTAAAAGAATAGATA
ATTGAGATATAATATCGTATGATCGGACGGCTGTGATCTGATGCCATCGGATGGGCACGTGTCAGAAGTGTACTCCAGGTATCAT
TCTCTCTGCCTCATGACATCAGCCGATGGTACCCTCGATACGGCGCCGGCAAGGCTCATATAATCCATTCAATTTATGTTTTTTCTG
CTAGCAAATAGATTTGCCATTGATTTGATACTCTCAATAATATTATCCGATTAAGGTATCATCCATATTGTTCAATATAATTTAAGC
ATATAACTGTTTCCAGATTAAACAATATAATTTATAAGAGCAACTGTAATACTTTACTTTAAAAGTTTTCAAATCAGAATCTCTTTT
CTTTTTCTACAAATCTGGAAACAATTTAATCCATATTGTTTTTGTTTTTTTTACCTTCTCATATTTAGATGCTATTTATTATTGTGACA
CAAGCTTAGTTTAATTTCTTGTTGGTTACATTTTTTTTTCTAAATACACAAATATTATATGTTTAATATTATTTCACACATTTCTTAGA
AGAAATCTAAATACATGAAATTATATTTATTAAAAAGAAGTCTAAACTATATATGTATTTAAATGGAATTTTAAATATGGGTAGT
AATATAGAAACACCATTGATATATTAGAGTATGTGCCGGTGCCGTATACATACTGTTGTATTATAGTAACCTATATATGGTATACC
TAGTCAATGTGTACGGTATTTTTCATATGTATTAGTTGTGAACCTCCACAGCATGTTAGACTTAGGAATTAATTATAACTGAACCA
GAACTTTGAGTCTAATTATACCCCTGCCGTTTTATACAACTTTGAATATTGATTCCAGTGGCTAATTTAAATAAGTTAAAGGAAAC
AAAAACTGCAAGTAAGTGAAAAATACACAATTGTAAGTAAATGTTAGATATTTAATTTATTTATAGCTAAATCATGAACAAGTTC
GTATAAGATCTAGATATATGTACCATATACCTTTTCTATGCCCACTATATATACTTACTACACATAATATATAAACCAACCAAAAT

ATTTGAATAGTTAATAACTATGATACACGTTTAGGACAAATAAAATTAAAAAATAGGAGATCCCATTTCTCGCCCTTGTAGGCTT
ACGCTATAAATTGGACTGTCCCCTTCTCATTTCATTACCAAAGTAAAGAACAGTTTCTATATTTCTCT

AP1 1895 bp

CTTGGGATGTTGTCTTCAAGGCCACGAGCTTAGATTCTTTTAGTTTTGCTCAATTTGTTAAGTTTCTACTTTTCCTTTTGTTGCTTACT
ACTTTTGCTCATGATCTCCATATACATATCATACATATATATAGTATACTATCTTTAGACTGATTTCTCTATACACTATCTTTTAACT
TATGTATCGTTTCAAAACTCAGGACGTACATGTTTAAATTTGGTTATATAACCACGACCATTTCAAGTATATATGTCATACCATAC
CAGATTTAATATAACTTCTATGAAGAAAATACATAAAGTTGGATTAAAATGCAAGTGACATCTTTTTAGCATAGGTTCATTTGGC
ATAGAAGAAATATATAACTAAAAATGAACTTTAACTTAAATAGATTTTACTATATTACAATTTTTTCTTTTTACATGGTCTAATTTA
TTTTTCTAAAATTAGTATAATTGTTGTTTTGATGAAACAATAATACCGTAAGCAATAGTTGCTAAAAGATGTCCAAATATTTATAA
ATTACAAAGTAAATCAAATAAGGAAGAAGACACGTGGAAAACACCAAATAAGAGAAGAAATGGAAAAAACAGAAAGAAATTT
TTTAACAAGAAAAATCAATTAGTCCTCAAACCTGAGATATTTAAAGTAATCAACTAAAACAGGAACACTTGACTAACAAAGAAA
TTTGAAACGTGGTCCAACTTTCACTTAATTATATTGTTTTCTCTAAGGCTTATGCAATATATGCCTTAAGCAAATGCCGAATCTGTT
TTTTTTTTTTTTGTTATTGGATATTGACTGAAAATAAGGGGTTTTTTCACACTTGAAGATCTCAAAAGAGAAAACTATTACAACGG
AAATTCATTGTAAAAGAAGTGATTAAGCAAATTGAGCAAAGGTTTTTATGTGGTTTATTTCATTATATGATTGACATCAAATTGTA
TATATATGGTTGTTTTATTTAACAATATATATGGATATAACGTACAAACTAAATATGTTTGATTGACGAAAAAAAATATATGTATG
TTTGATTAACAACATAGCACATATTCAACTGATTTTTGTCCTGATCATCTACAACTTAATAAGAACACACAACATTGAACAAATCT
TTGACAAAATACTATTTTTGGGTTTGAAATTTTGAATACTTACAATTATTCTTCTCGATCTTCCTCTCTTTCCTTAAATCCTGCGTAC
AAATCCGTCGACGCAATACATTACACAGTTGTCAATTGGTTCTCAGCTCTACCAAAAACATCTATTGCCAAAAGAAAGGTCTATT
TGTACTTCACTGTTACAGCTGAGAACATTAAATATAATAAGCAAATTTGATAAAACAAAGGGTTCTCACCTTATTCCAAAAGAAT
AGTGTAAAATAGGGTAATAGAGAAATGTTAATAAAAGGAAATTAAAAATAGATATTTTGGTTGGTTCAGATTTTGTTTCGTAGAT
CTACAGGGAAATCTCCGCCGTCAATGCAAAGCGAAGGTGACACTTGGGGAAGGACCAGTGGTCCGTACAATGTTACTTACCCAT
TTCTCTTCACGAGACGTCGATAATCAAATTGTTTATTTTCATATTTTTAAGTCCGCAGTTTTATTAAAAAATCATGGACCCGACATT
AGTACGAGATATACCAATGAGAAGTCGACACGCAAATCCTAAAGAAACCACTGTGGTTTTTGCAAACAAGAGAAACCAGCTTTA
GCTTTTCCCTAAAACCACTCTTACCCAAATCTCTCCATAAATAAAGATCCCGAGACTCAAACACAAGTCTTTTTATAAAGGAAAG
AAAGAAAAACTTTCCTAATTGGTTCATACCAAAGTCTGAGCTCTTCTTTATATCTCTCTTGTAGTTTCTTATTGGGGGTCTTTGTTTT
GTTTGG
